# Casein Kinase 1 Epsilon Regulates Glioblastoma Cell Survival

**DOI:** 10.1038/s41598-018-31864-x

**Published:** 2018-09-11

**Authors:** Robin T. Varghese, Sarah Young, Lily Pham, Yanping Liang, Kevin J. Pridham, Sujuan Guo, Susan Murphy, Deborah F. Kelly, Zhi Sheng

**Affiliations:** 1 Virginia Tech Carilion Research Institute, Roanoke, VA 24016 United States; 2 Department of Internal Medicine, Virginia Tech Carilion School of Medicine, Roanoke, VA 24016 United States; 3 Graduate Program in Translational Biology, Medicine, and Health, Virginia Tech, Blacksburg, VA 24061 United States; 4 Department of Biological Sciences, College of Sciences at Virginia Tech, Blacksburg, VA 24061 United States; 5 Faculty of Health Science, Virginia Tech, Blacksburg, VA 24061 United States; 6 Present Address: Edward Via College of Osteopathic Medicine, Blacksburg, VA 24060 USA

## Abstract

Glioblastoma is the most common malignant brain cancer with a dismal prognosis. The difficulty in treating glioblastoma is largely attributed to the lack of effective therapeutic targets. In our previous work, we identified casein kinase 1 ε (CK1ε, also known as CSNK1E) as a potential survival factor in glioblastoma. However, how CK1ε controls cell survival remains elusive and whether targeting CK1ε is a possible treatment for glioblastoma requires further investigation. Here we report that CK1ε was expressed at the highest level among six CK1 isoforms in glioblastoma and enriched in high-grade glioma, but not glia cells. Depletion of CK1ε remarkably inhibited the growth of glioblastoma cells and suppressed self-renewal of glioblastoma stem cells, while having limited effect on astrocytes. CK1ε deprivation activated β-catenin and induced apoptosis, which was further counteracted by knockdown of β-catenin. The CK1ε inhibitor IC261, but not PF-4800567, activated β-catenin and blocked the growth of glioblastoma cells and glioblastoma stem cells. Congruently, IC261 elicited a robust growth inhibition of human glioblastoma xenografts in mice. Together, our results demonstrate that CK1ε regulates the survival of glioblastoma cells and glioblastoma stem cells through β-catenin signaling, underscoring the importance of targeting CK1ε as an effective treatment for glioblastoma.

## Introduction

Glioblastoma (GBM) is the most common form of primary malignant cancer in the central nervous system^1^. Standard treatments after diagnosis include surgical removal of the bulk tumor, radiation, and chemotherapy. Despite such an aggressive course of treatment, the median survival time of GBM patients has only been extended from 12 months to 14.6 months^2^. Moreover, nearly 90% of GBM patients, if they live longer than two years, develop and succumb to recurrent tumors^3,4^. As such, the percentage of GBM patients with 5-year survival is only 5.5%^1^. Thus, there is an unmet need of effective treatments for this deadly disease.

To search for novel therapeutic targets for GBM, we performed a loss-of-function screen in U87MG human GBM cells using a library of short hairpin RNAs (shRNAs) targeting human kinases^5^. Protein kinases are excellent therapeutic targets as they are often amplified or mutated in cancer and are well fit for structure-based drug design of small molecule inhibitors^6^. From approximately 4,000 shRNAs that target 784 human kinase genes, 20 kinases were identified as potentially important survival factors. One candidate, casein kinase 1 ε (CK1ε or CSNK1E), has drawn our attention because multiple shRNAs of CK1ε were found in the screen and the role of CK1ε in GBM remains to be elucidated.

CK1ε is a member of the CK1 gene family, which consists of six isoforms (α, γ1, γ2, γ3, δ, and ε). The differential expression levels of CK1 genes in tissues and their capacity to activate downstream targets result in tissue-specific function of each CK1 isoform^7^. While CK1ε has been previously reported as a key modulator of circadian rhythm^8^, its role in cancer cell survival has just emerged. For example, pharmacological inhibition or shRNA-mediated ablation of CK1ε impedes the growth or blocks the survival of pancreatic cancer, sarcoma, breast cancer, colorectal cancer, ovarian cancer, and leukemic cells^9–14^. However, how CK1ε regulates cancer cell survival is not well understood, partly because of the lack of substrate specificity of CK1 genes^15^. It has been reported that CK1ε promotes disease progression in some cancers through different targets such as MYC (MYC proto-oncogene, bHLH transcription factor), AKT (v-akt murine thymoma viral oncogene homolog), or β-catenin (catenin beta 1, also known as CTNNB1)^11,14,16^. Nonetheless, the mechanism underlying CK1ε-regulated cell survival in GBM has not yet been defined and the therapeutic potential of targeting CK1ε requires further investigation.

Here we report that CK1ε was barely detected in glia cells, but highly enriched in GBM. Knockdown of CK1ε induced significant inhibition of cell viability in an array of GBM cell lines, while having a negligible effect on the survival of astrocytes and HEK293 cells. CK1ε deficiency activated β-catenin and, in turn, induced apoptosis and growth inhibition. Moreover, blocking CK1ε diminished the capacity of GBM stem cells (GSCs) to divide. The CK1ε inhibitor IC261, but not PF-4800547, activated β-catenin and mitigated the growth of GBM cells and GSCs *in vitro* and *in vivo*. Our results demonstrate that CK1ε promotes GBM cell survival though attenuating the activity of β-catenin and targeting CK1ε represents an appealing therapeutic option for GBM.

## Results

### CK1ε is highly expressed in GBM

**Figure 1:**
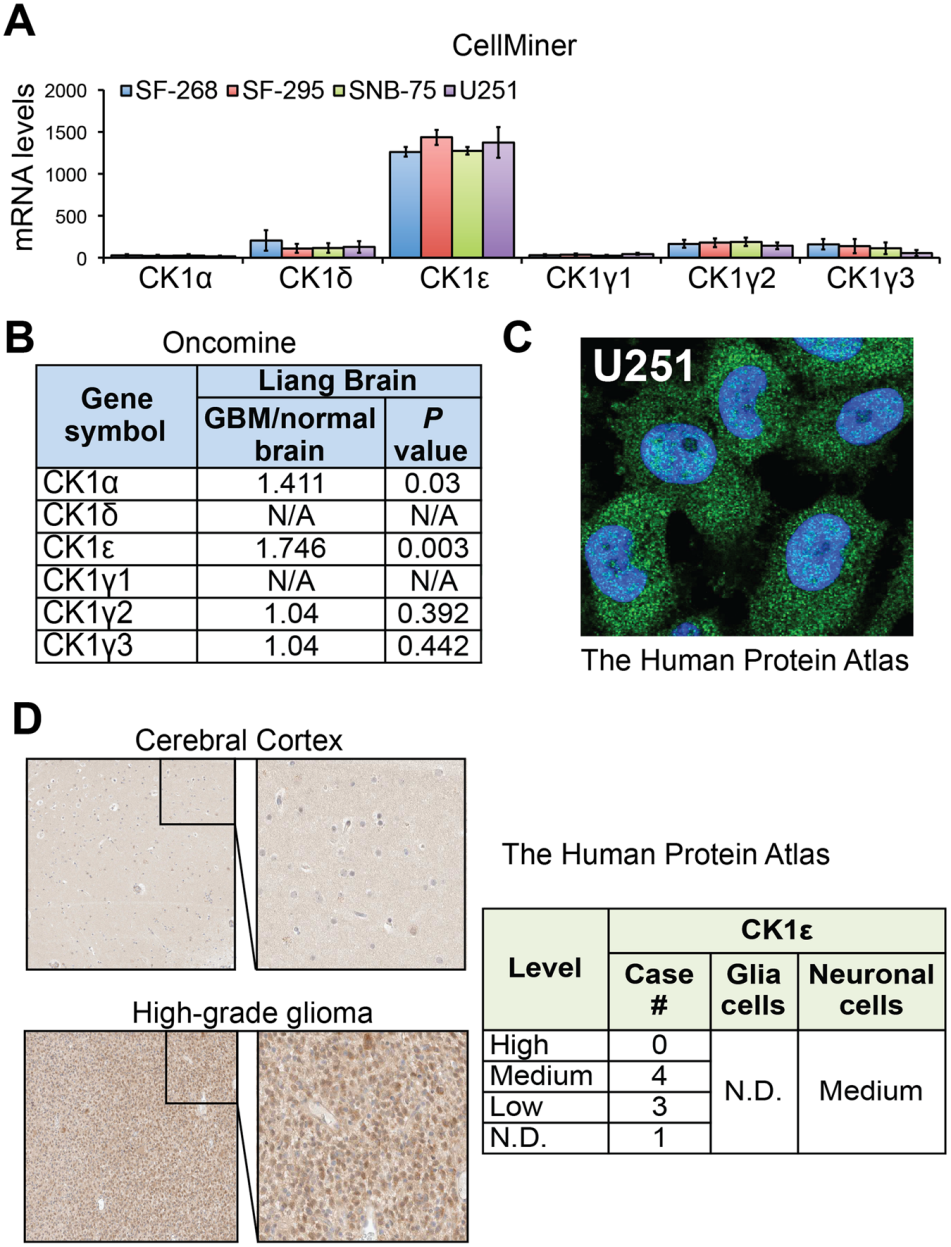
CK1ε is highly expressed in GBM. (**A**) mRNA levels of CK1 genes in four GBM cell lines. Data was retrieved from the CellMiner database. The arbitrary copy numbers are shown. Error bars represent standard deviations from four different sets of data. (**B**) mRNA levels of CK1 genes in normal and GBM tissues. Data was retrieved from the Oncomine database. Fold changes of mRNAs in GBM tissues over mRNAs in normal brain tissues are shown. *P* values determine the statistical significance of mRNA difference between GBM and normal brain tissues. N/A: not available. (**C**) Immunofluorescence analysis of CK1ε in U251 cells. Green: CK1ε; Blue: nuclei. Data were from The Human Protein Atlas. (**D**) Immunohistochemical analyses of CK1ε in normal brain tissues and specimens of high-grade glioma. Data were from the Human Protein Atlas. N.D.: not detected.

We first monitored the expression levels of CK1 isoforms through querying online databases. Based on gene expression data from CellMiner, CK1ε mRNA was expressed at a significantly higher level than other CK1 isoforms in four GBM cell lines SF-268, SF-295, SNB-75, and U251 (Fig. 1A). Data from a cDNA microarray study (named Liang Brain) in the Oncomine database showed that fold changes of CK1α, CK1ε, CKγ2, and CKγ3 in GBM tissues over normal brain tissues were 1.411, 1.746, 1.04, and 1.04, respectively (Fig. 1B). CK1ε levels were higher than other isoforms in GBM patient specimens, consistent with the results in GBM cell lines. To determine the localization and expression levels of CK1ε protein, we queried The Human Protein Atlas database. CK1ε proteins primarily localized to cytoplasm and nucleus in U251 GBM cells (Fig. 1C). Compared to normal cerebral cortex and glia cells, CK1ε proteins were expressed at high levels in high-grade glioma (Fig. 1D). While sample size in some results was small, these data suggest that CK1ε is highly expressed in GBM.

### CK1ε is important for GBM cell survival

**Figure 2:**
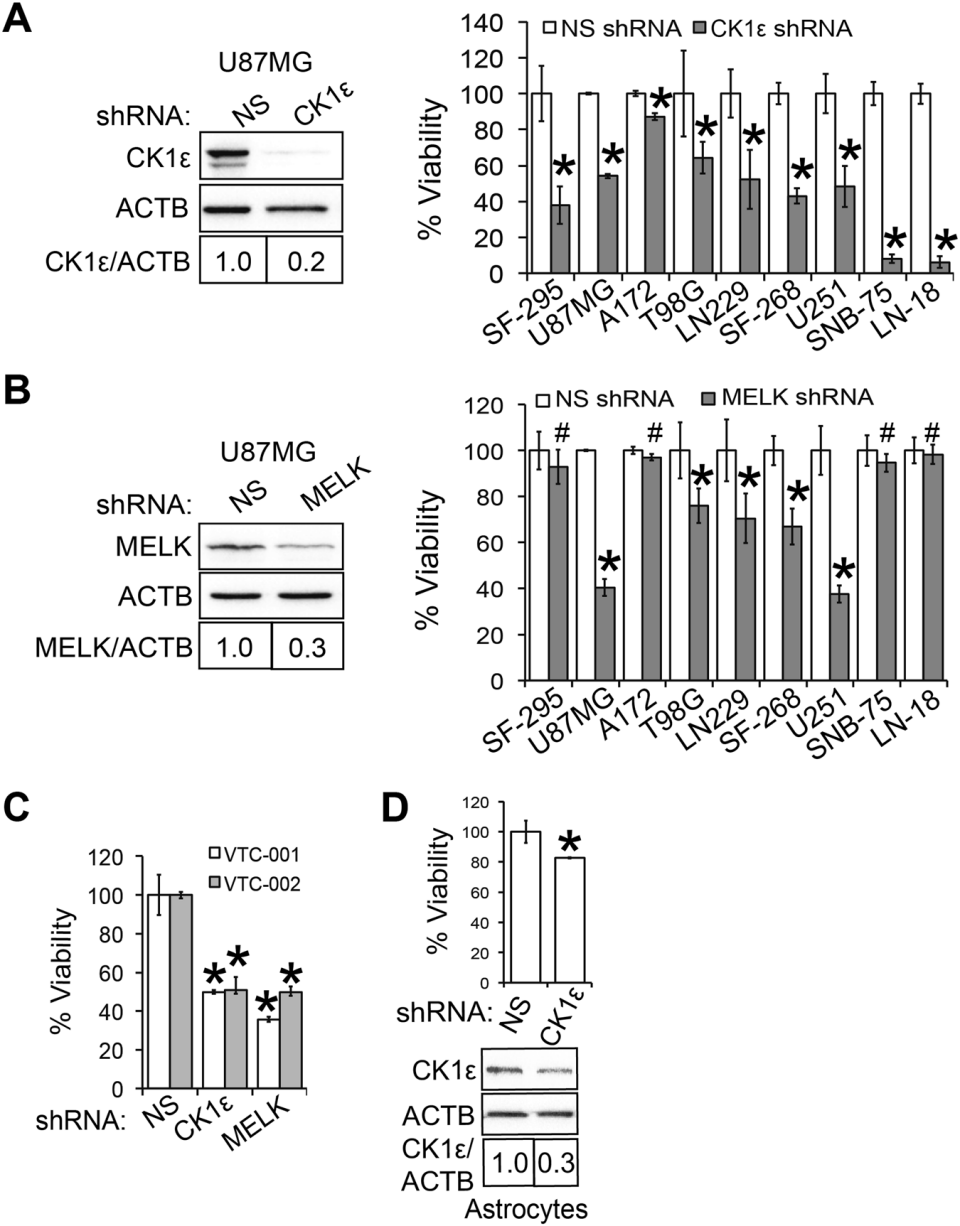
CK1ε is important for GBM cell survival. (**A**) Knockdown of CK1ε in nine GBM cell lines. GBM cells were treated with non-silencing (NS) or CK1ε shRNA. Protein levels of CK1ε in U87MG cells are shown in the left panel. ACTB (β-actin) is the loading control. Band intensities were quantified using Image J. The viability of GBM cell lines was determined by the MTS viability assay and is shown in the right panel. (**B**) Knockdown of MELK in nine GBM cell lines. Left panel: immunoblotting of MELK in U87MG cells. Right panel: cell viability. (**C**) Viability of primary GBM cells upon depletion of CK1ε or MELK. (**D**) Knockdown of CK1ε in astrocytes. Top panel: cell viability; bottom panel: immunoblotting of CK1ε. Full length blots were presented in supplemental materials. *P < 0.05; ^#^*P* > 0.05.

Next, we sought to verify that CK1ε, a candidate survival kinase gene from our previous RNA interference screen, is important for GBM cell survival through knocking down CK1ε in nine GBM cell lines. As indicated by CK1ε immunoblotting (Fig. 2A, left panel and Fig. S1), CK1ε shRNA decreased CK1ε protein levels by 3-10-fold in nine GBM cell lines tested. Upon CK1ε depletion, the viability of SF-295, U87MG, LN229, SF-268, and U251 cells dropped to less than 60% and that of SNB-75 and LN-18 was even below 10% (Fig. 2A, right panel). These cell lines are hereafter designated as CK1ε shRNA-responsive GBM cells. However, the inhibitory effect on the viability of A172 or T98G cells was only modest (>60%), so they are CK1ε shRNA-nonresponsive GBM cells. In our previous report^5^, we also identified MELK (maternal embryonic leucine zipper kinase) as a survival kinase gene. Because MELK was a known survival factor in GBM^17^, we herein used this candidate kinase as a control to compare the effect of CK1ε and MELK on GBM cell survival. We found that MELK deficiency (Fig. 2B, left panel and Fig. S2) only mitigated the viability of U87MG and U251 cells (<60%), while having no or modest effect on the remaining GBM cell line (Fig. 2B, right panel). Our results suggest that CK1ε is important for the survival of multiple GBM cell lines.

To corroborate the above results, we determined the viability of two primary GBM cell lines (VTC-001 and VTC-002), which were recently isolated from patient specimens^18^. shRNAs of CK1ε or MELK significantly inhibited the viability of primary VTC-001 and VTC-002 cells (Fig. 2C), consistent with the results from GBM cell lines (Fig. 2A,B). Because CK1ε was more highly expressed in GBM tissues than in normal brain tissues (Fig. 1B,D), we hypothesized that this kinase was not important for the viability of normal cells. As expected, CK1ε deficiency only induced a slight or no decrease of cell viability in astrocytes (Fig. 2D) and HEK293 cells (Fig. S3). Collectively, our results demonstrate that CK1ε is a pivotal survival factor for GBM.

### Loss of CK1ε induces apoptosis and growth inhibition through activating β-catenin

**Figure 3:**
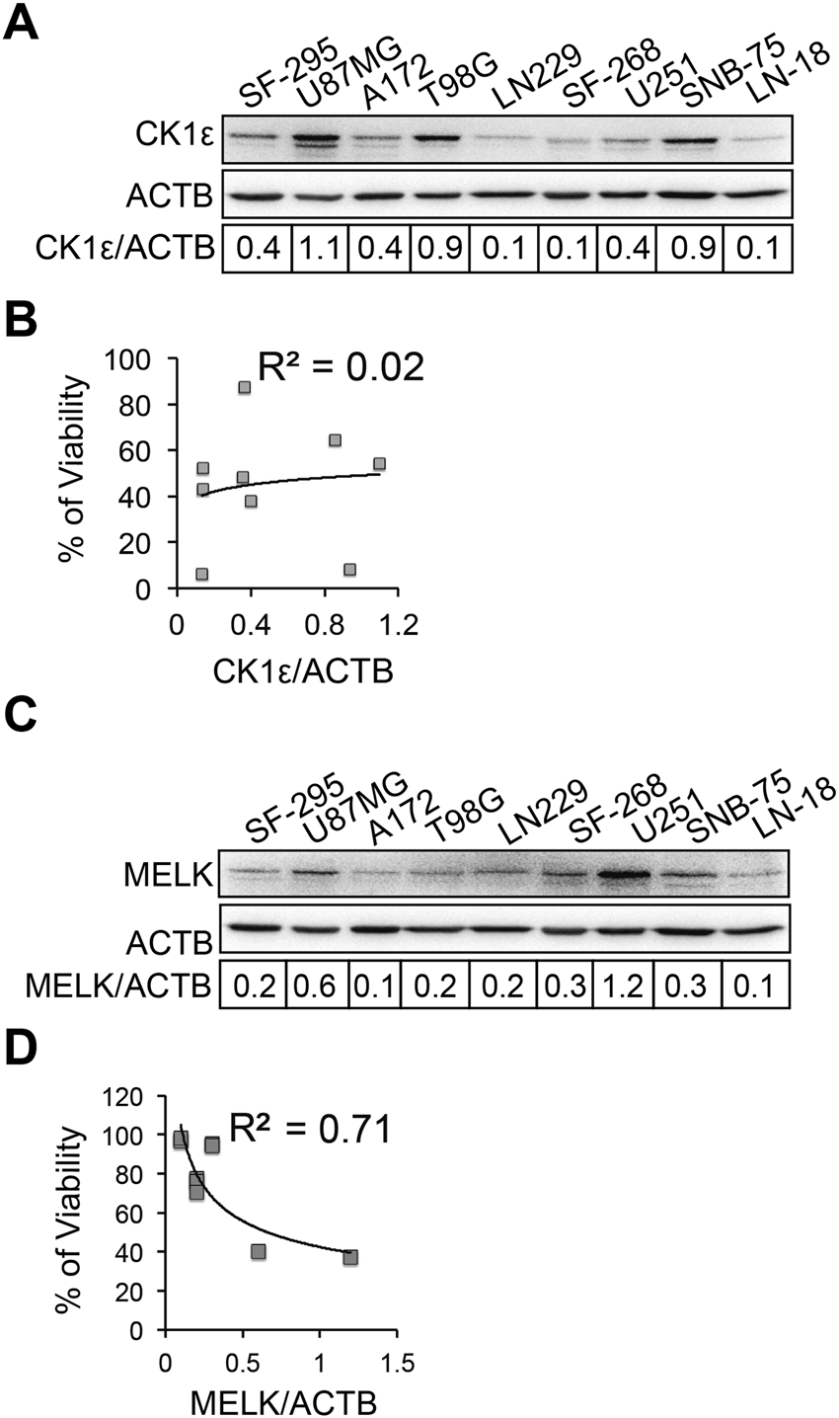
Cell responses to CK1ε depletion do not correlate with levels of CK1ε. (**A**) Immunoblotting of CK1ε in GBM cell lines. ACTB (β-actin) is the loading control. Band intensities were quantified using Image J. (**B**) Correlation of CK1ε protein levels and viability of CK1ε-deficient GBM cell lines. A linear regression model was used to determine the correlation between CK1ε levels and GBM cell viability upon CK1ε depletion (Fig. 2A, right panel). R square is the coefficient of determination. (**C**) Immunoblotting of MELK in GBM cell lines. (**D**) Correlation of MELK protein levels and viability of GBM cell lines. Full length blots were presented in supplemental materials.

To determine whether responses of GBM cell lines to CK1ε depletion depend on levels of CK1ε protein, we performed immunoblotting of CK1ε in GBM cell lines. We found that U87MG, T98G, and SNB-75 expressed higher levels of CK1ε than other cell lines (Fig. 3A). To determine the correlation between cell viability affected by CK1ε depletion and levels of CK1ε, we utilized a linear regression model to reveal the determination coefficient R^2^, which indicates how strong the correlation is. We found that levels of CK1ε protein had no association with the responsiveness of GBM cells to CK1ε depletion (R^2^ = 0.02; Fig. 3B). In contrast, high levels of MELK protein (Fig. 3C) positively correlated with MELK shRNA-induced growth inhibition (R^2^ = 0.71; Fig. 3D).

**Figure 4:**
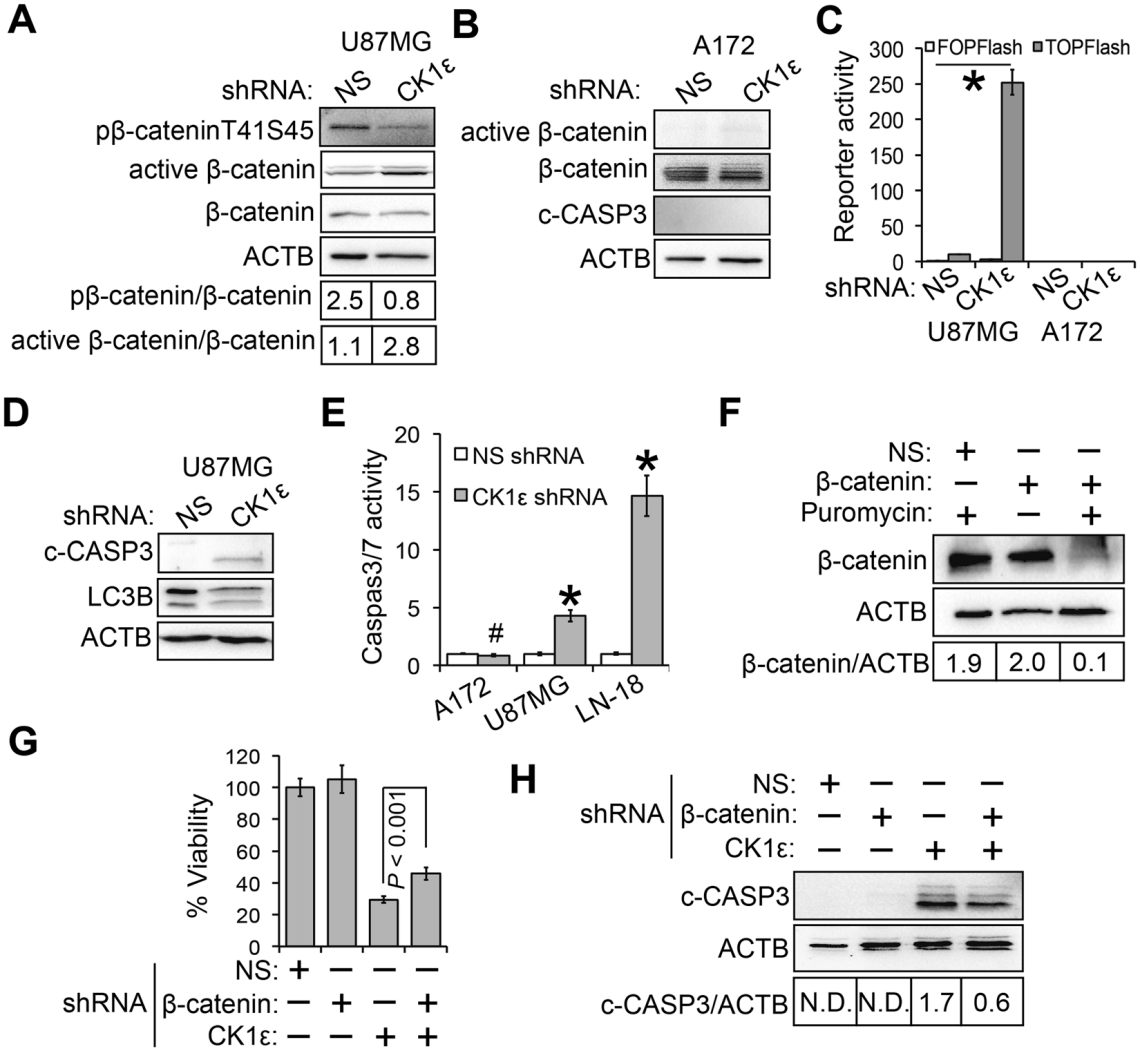
CK1ε depletion induces apoptosis and growth inhibition through activating β-catenin. (**A**) β-catenin signaling in CK1ε-deficient U87MG cells. Phosphorylated, active, and total β-catenin was assessed using immunoblotting. ACTB (β-actin) is the loading control. Band intensities were quantified using Image J. (**B**) β-catenin signaling in CK1ε-deficient A172 cells. (**C**) Luciferase reporter assay. The TOPFlash plasmid harbors TCF/LEF binding sites and responds to β-catenin activation. FOPFlash contains mutated TCF/LEF binding sites and does not respond to β-catenin activation. (**D**) Immunoblotting of cleaved caspase 3 (c-CASP3, an apoptosis marker) and LC3B (an autophagy marker). (**E**) Caspase 3/7 activity assay. (**F**) Knockdown of β-catenin. U87MG cells were transduced with viruses of NS shRNA or β-catenin shRNA. (**G**) Viability of U87MG cells treated with CK1ε shRNA and/or β-catenin shRNA. (**H**) Immunoblotting of c-CASP3 in U87MG cells upon depletion of CK1ε and/or β-catenin. Error bars represent standard deviations from three independent experiments. Full length blots were presented in supplemental materials. *P < 0.05; ^#^*P* > 0.05. N.D.: not detected.

These results led us to hypothesize that the activity, rather than the expression level, of CK1ε is important for GBM cell survival. To test this hypothesis, we measured β-catenin activity in CK1ε-deficient U87MG cells, because β-catenin is one of the major signaling pathways regulated by CK1 genes in cancer cells^19^ and is important for gliomagenesis^20,21^. In the canonical WNT/β-catenin signaling pathway, CK1ε phosphorylates β-catenin at threonine 41 and serine 45 together with GSK3β (glycogen synthase kinase 3 β), thereby facilitating β-catenin degradation when WNT ligands are absent^22,23^. Upon stimulation by WNT ligands, CK1ε facilitates β-catenin activation^19,24–26^. Hence, whether CK1ε inhibits or activates β-catenin depends upon WNT ligands. Herein, we assumed that CK1ε inactivates β-catenin in GBM cells due to the lack of WNT ligands. Consistent with our expectation, pβ-cateninT41S45 was reduced by approximately 3-fold in U87MG cells upon depletion of CK1ε, coinciding with a more than 2-fold increase of non-phosphorylated/active β-catenin (Fig. 4A). In addition to CK1ε shRNA-responsive U87MG cells, we also monitored β-catenin activity in CK1ε shRNA-nonresponsive A172 cells (Fig. 2A, right panel). The ratios of active β-catenin/β-catenin remained unchanged upon depletion of CK1ε in CK1ε shRNA-nonresponsive A172 cells (Fig. 4B). To confirm that CK1ε depletion does activate β-catenin signaling, we monitored the activity of T-cell factor/lymphoid enhancer factor (TCF/LEF), a major downstream target of activated β-catenin in the nucleus^19,24–26^. The TOPFlash reporter plasmid contains TCF/LEF binding sites, which are mutated and inactivated in the FOPFlash plasmid^27^. The reporter activity of both TOPFlash and FOPFlash was low in NS(non-silencing)-shRNA-treated CK1ε shRNA-responsive U87MG and CK1ε shRNA-nonresponsive A172 cells, as expected (Fig. 4C). Upon treatment of CK1ε shRNA, TOPFlash was activated in U87MG cells, but not in A172 cells. FOPFlash was not activated by CK1ε deprivation. These results, consistent with those shown in Fig. 4A,B, demonstrate that CK1ε suppresses β-catenin activity in CK1ε shRNA-responsive GBM cells, but not in CK1ε shRNA-nonresponsive GBM cells.

To gain more insights into the role of CK1ε in GBM cell survival, we measured cell death in CK1ε-deficient GBM cells. We found that c-CASP3 (cleaved caspase 3, an apoptosis marker) significantly increased, whereas levels of LC3B (microtubule-associated proteins 1A/1B light chain 3B; an autophagy marker) decreased, upon CK1ε depletion in CK1ε shRNA-responsive U87MG cells (Fig. 4D). In contrast, no apoptosis was detected in CK1ε shRNA-nonresponsive A172 cells (Fig. 4B, panel c-CASP), congruent with the results shown in Fig. 2A. By using the caspase 3/7 activity assay, we found that CK1ε shRNA activated apoptosis in U87MG and LN-18 cells, but not in A172 cells (Fig. 4E). These results, together with results described previously, demonstrate that CK1ε inhibits apoptosis and promotes cell survival in GBM.

Based upon above results, we posited that activated β-catenin was required for apoptosis and growth inhibition induced by CK1ε deficiency. To test this hypothesis, we knocked down both CK1ε and β-catenin in CK1ε shRNA-responsive U87MG cells. Immunoblotting of β-catenin showed that the β-catenin shRNA induced an approximately 20-fold decrease of β-catenin protein (Fig. 4F) when U87MG cells were selected by puromycin (the β-catenin shRNA construct contains a puromycin-resistant gene). Deprivation of β-catenin decreased the levels of growth inhibition and apoptosis induced by CK1ε deficiency (Fig. 4G,H). In contrast, β-catenin shRNA alone had no effect on cell viability and apoptosis. Taken together, CK1ε deprivation induces apoptosis and inhibits cell growth through activating β-catenin signaling in CK1ε shRNA-responsive GBM cells.

### CK1ε regulates self-renewal of GSCs

**Figure 5:**
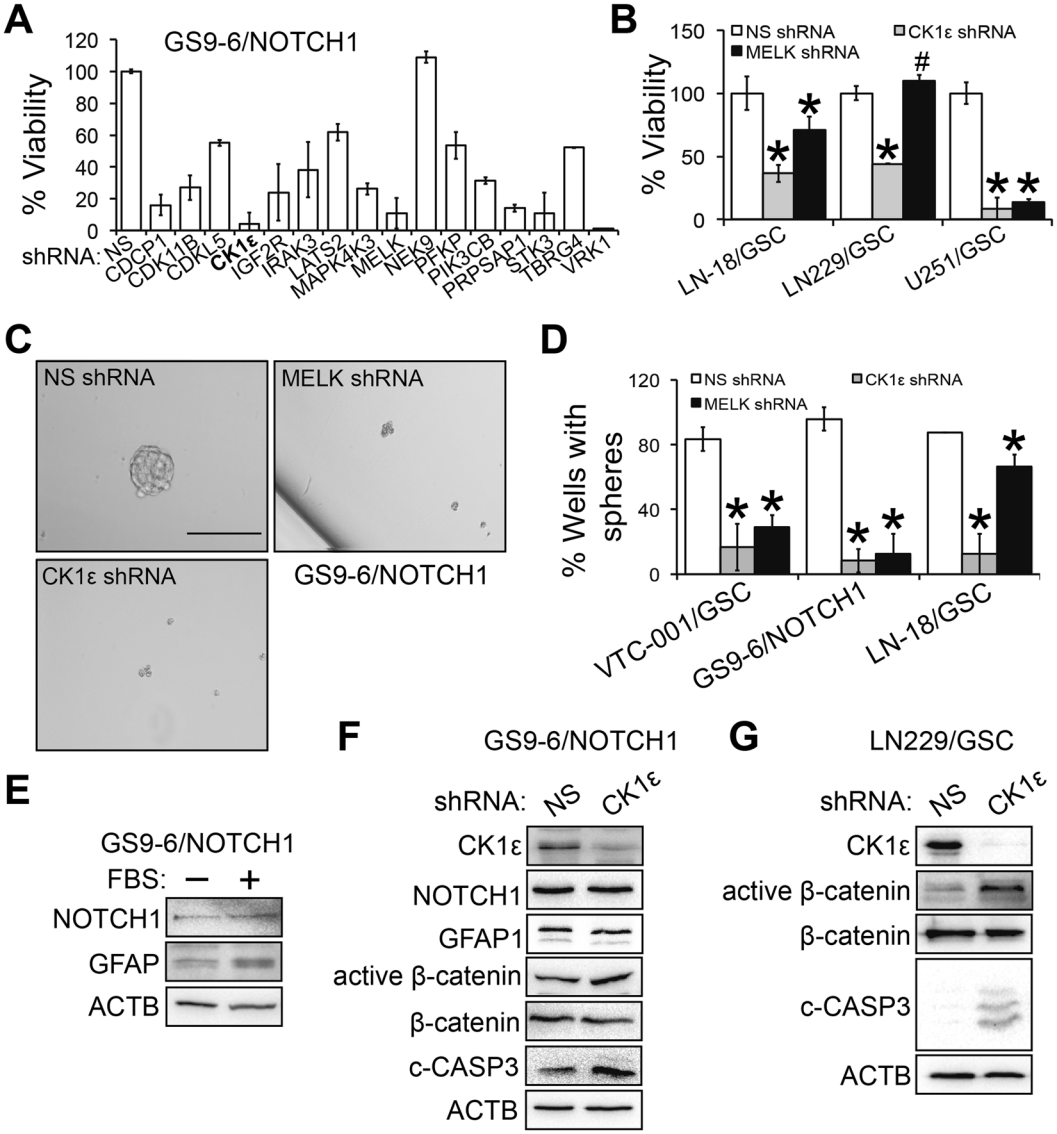
CK1ε regulates β-catenin activity and self-renewal of GSCs. (**A**) Viability of GS9-6/NOTCH1 GSCs upon depletion of survival kinase genes. GS9-6/NOTCH1 cells were transduced with viruses of NS shRNA or shRNAs of individual kinase genes from our previous RNA interference screen (ref.^5^). (**B**) Viability of LN-18/GSC, LN229/GSCs, and U251/GSC upon depletion of CK1ε or MELK. (**C**) Images of GS9-6/NOTCH1 spheres treated with NS, CK1ε, or MELK shRNA. Scale bar: 25 μm. (**D**) Self-renewal assay of VTC-001/GSC, GS9-6/NOTCH1, and LN-18/GSC treated with NS, CK1ε, or MELK shRNA. Percentages of wells with spheres represent capabilities of GSCs to self-renew. (**E**) Differentiation of GS9-6/NOTCH1 cells. Cells were treated with fetal bovine serum (FBS) to induce differentiation, which was indicated by elevated levels of GFAP (an astrocyte marker). (**F**) Immunoblotting of active β-catenin, β-catenin, c-CASP3, NOTCH1, and GFAP in CK1ε-deficient GS9-6/NOTCH1 cells. (**G**) Immunoblotting of active β-catenin, β-catenin, or c-CASP3 in CK1ε-deficient LN229/GSCs. Full length blots were presented in supplemental materials. *P < 0.05; ^#^*P* > 0.05.

The heterogeneity among different GBM tumors or within a given tumor makes GBM difficult to treat. For example, GBM stem cells (GSCs), a small population of tumor cells that are resistant to current therapies, are thought to be the culprit of GBM formation and disease progression^28,29^. The identification of survival kinase genes in U87MG cells^5^ prompted us to further investigate the role of these kinases in GSCs. In GS9-6/NOTCH1 GSCs we characterized recently^30^, 11 shRNAs of survival kinase genes robustly reduced cell viability (<50%; Fig. 5A), among which CK1ε shRNA (highlighted in bold) was one of the candidates with the strongest cytotoxicity. To test whether this inhibitory effect was cell line specific, we knocked down CK1ε in LN-18/GSC, LN229/GSC, and U251/GSC recently established in our laboratory^31^. CK1ε depletion induced a remarkable growth inhibition in these GSCs (Fig. 5B). However, ablation of MELK, a known survival factor of GSC^17^, only effectively blocked the growth of U251/GSCs and had no or limited effect on the viability of LN-18/GSCs and LN229/GSCs.

GSCs are different from other differentiated tumor cells because GSCs can self-renew (copy themselves) and differentiate (convert into differentiated cells)^32^. We then hypothesized that CK1ε regulates GSCs’ self-renewal and/or differentiation. To test this hypothesis, we first monitored GSC self-renewal using the sphere formation assay. Knockdown of CK1ε or MELK blocked sphere formation in GS9-6/NOTCH1 GSCs and VTC-001/GSCs (Fig. 5C,D), the latter of which is a patient-derived GSC line from our previous report^31^. CK1ε shRNA inhibited the ability of LN-18/GSCs to self-renew, whereas MELK shRNA failed to do so (Fig. 5D). These results are consistent with the differential effect of CK1ε or MELK shRNA on the viability of LN-18/GSCs (Fig. 5B) and parental CK1ε shRNA-responsive LN-18 cells (Fig. 2A,B). To test GSCs’ differentiation, we used an approach in our previous report^33^ to monitor serum-induced differentiation in GS9-6/NOTCH1 GSCs. As expected, serum treatment increased the levels of GFAP (glial fibrillary acidic protein, an astrocyte marker) in GS9-6/NOTCH1 GSCs (Fig. 5E). By contrast, depletion of CK1ε failed to induce differentiation of GS9-6/NOTCH1 GSCs, as manifested by no change of GFAP (Fig. 5F). Because β-catenin signaling is also important for the growth of stem cells^34^, we monitored β-catenin activity and apoptosis in CK1ε-deficient GSCs. Upon CK1ε deprivation, levels of both activated β-catenin and c-CASP3 significantly increased in GS9-6/NOTCH1 GSCs (Fig. 5F). Similar results were obtained in CK1ε-deficient LN229/GSCs (Fig. 5G). Hence, CK1ε determines the capability of GSCs to self-renew and regulates β-catenin activity in GSCs.

### The CK1ε inhibitor IC261 suppresses GBM growth *in vitro* and *in vivo*

**Figure 6:**
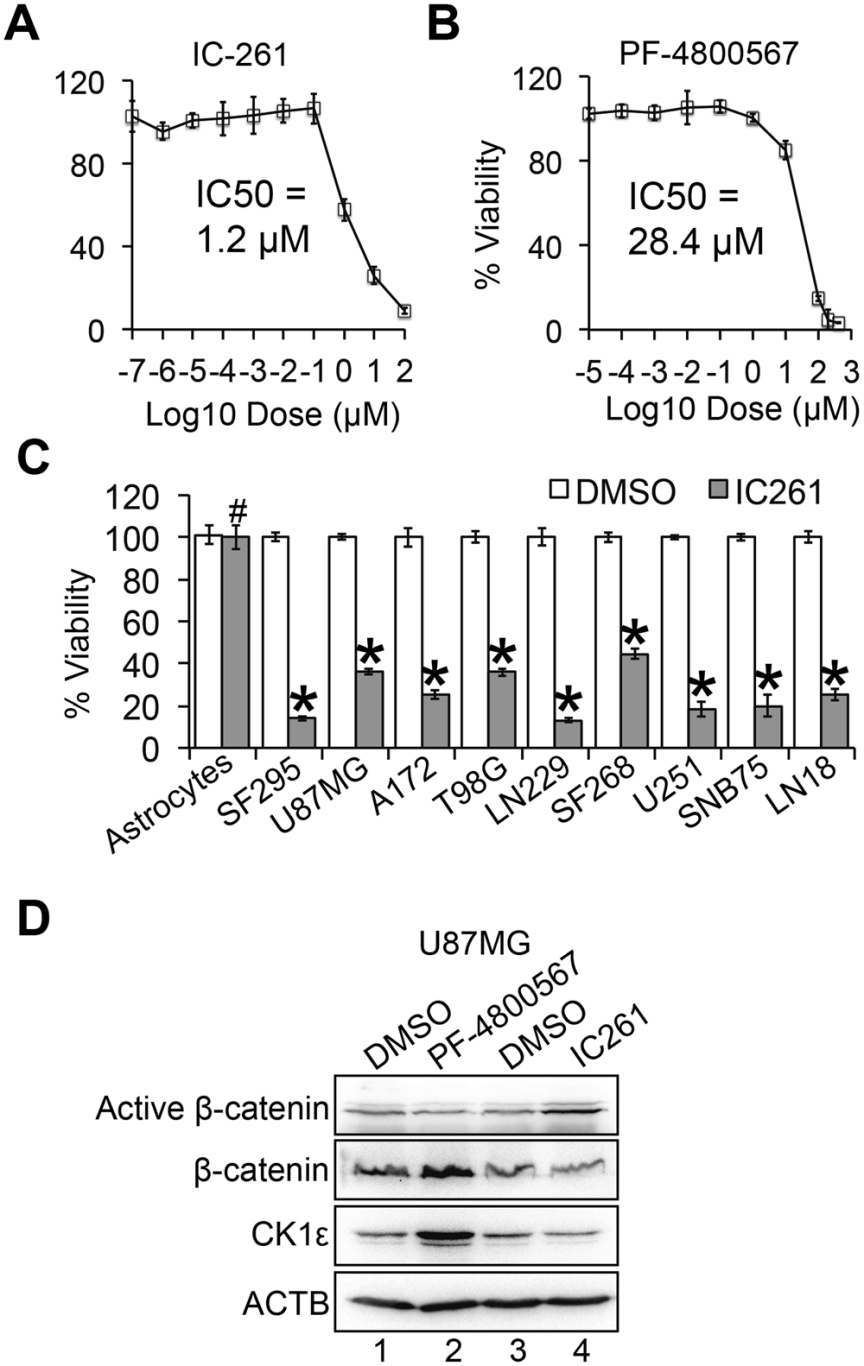
The CK1ε inhibitor IC261 blocks GBM cell growth with no effect on astrocytes. (**A**) IC50 of IC261 in U87MG cells. U87MG cells were treated with IC261 at various doses. Viability was determined using the MTS assay and IC50 was calculated using Prism. (**B**) IC50 of PF-4800567 in U87MG cells. (**C**) Cytotoxicity of IC261 in astrocytes and nine GBM cell lines. (**D**) Immunoblotting of CK1ε/β-catenin signaling in U87MG cells treated with IC261 or PF-4800567. Full length blots were presented in supplemental materials. *P < 0.05; ^#^*P* > 0.05.

To explore the therapeutic potential of targeting CK1ε in GBM, we utilized PF-4800567 and IC261, two CK1ε chemical inhibitors reported previously^35,36^. IC261 yielded a strong growth inhibition in CK1ε shRNA-responsive U87MG cells with an IC50 of 1.2 μM (Fig. 6A). In contrast, PF-4800567 had a modest effect on the viability of U87MG cells with an IC50 of approximately 28.4 μM (Fig. 6B). We also monitored the cytotoxicity of IC-261 in other GBM cell lines and astrocytes. Surprisingly, IC261 displayed similar growth inhibition in all nine GBM cell lines including CK1ε shRNA-nonresponsive T98G and A172 cells (Figs 2A and 6C). This discrepancy could be explained by the low specificity and selectivity of IC261 in inhibiting CK1ε because this inhibitor also blocks CK1δ^35,37^. Congruent with CK1ε shRNA, IC261 also had no effect on the viability of astrocytes (Fig. 6C). These results demonstrate that CK1ε inhibitors block tumor cell growth, while sparing normal astrocytes. Hence, targeting CK1ε becomes an appealing therapeutic approach for GBM.

PF-4800567 is reported as a selective inhibitor of CK1ε because it blocks CK1ε-controlled activation of circadian rhythm genes^36^. However, the cytotoxicity of PF-4800567 to GBM cells was not as strong as the non-selective CK1ε inhibitor IC261 (Fig. 6A,B). Given the important role of β-catenin in CK1ε-regulated cell survival (Fig. 4), we postulated that PF-4800567 and IC261 had different capabilities in activating β-catenin. Consistent with our expectation, PF-4800567 increased CK1ε protein levels but decreased the levels of active β-catenin (Fig. 6D, lanes 1–2), whereas IC261 significantly activated β-catenin with a concomitant decrease of CK1ε (Fig. 6D, lanes 3–4). Hence, while PF-4800567 prevents CK1ε from activating circadian rhythm genes^36^, this drug fails to activate β-catenin and is therefore not as potent as IC261. Future work should aim at identifying CK1ε selective inhibitors that activate β-catenin and induce apoptosis in GBM (see Discussion for details).

**Figure 7:**
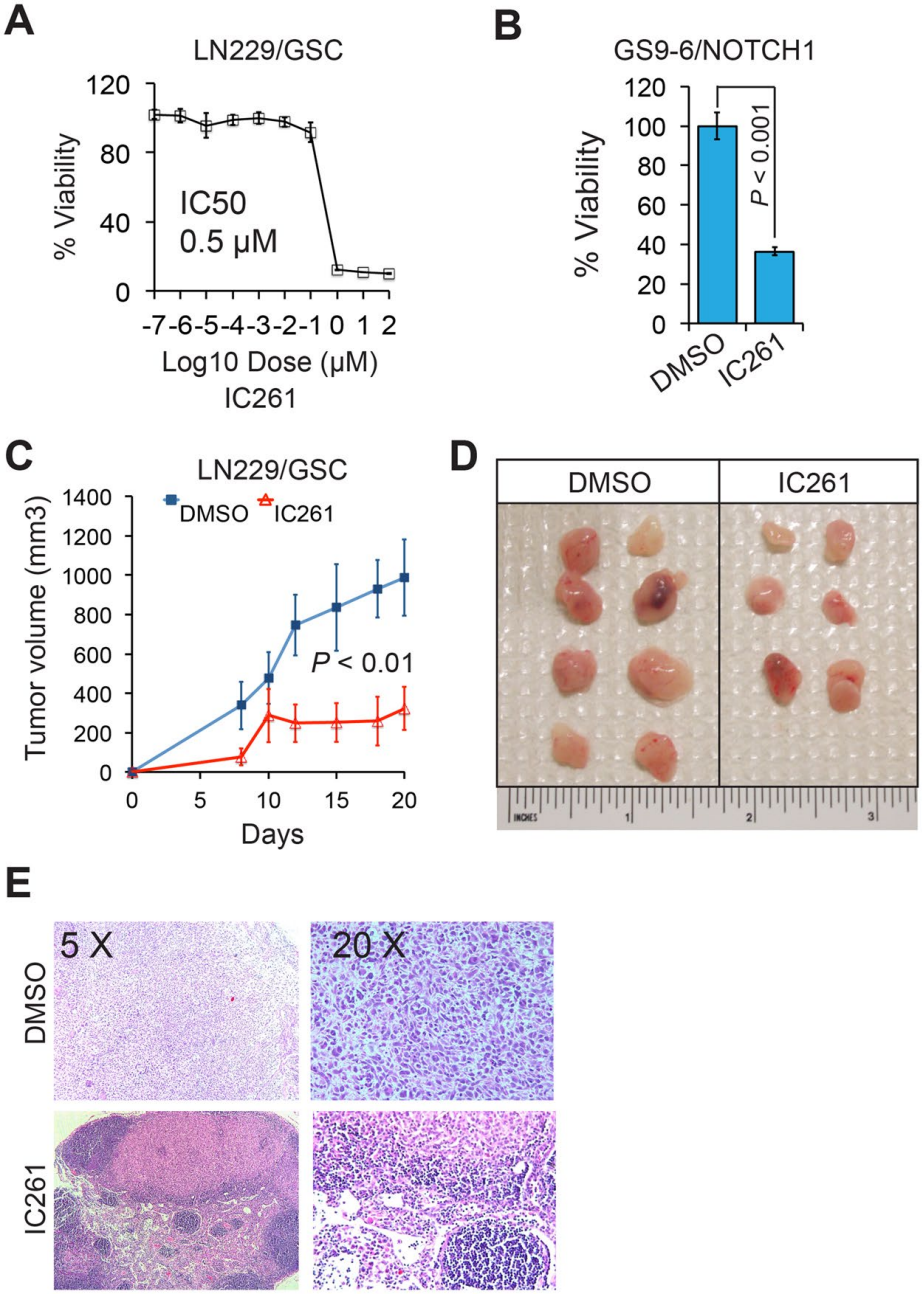
The CK1ε inhibitor IC261 blocks the growth GSCs *in vitro* and *in vivo*. (**A**) IC50 of IC261 in LN229/GSCs. (**B**) Viability of GS9-6/NOTCH1 treated with IC261. (**C**) Tumor growth in mice. LN229/GSCs were injected subcutaneously into immune-deficient mice followed by treatment of DMSO or IC261 (30 mg/kg). (**D**) Images of xenograft tumors at the end point. (**E**) Histological analysis of tumors.

To explore the therapeutic potential of CK1ε inhibitors in GSCs, we treated GSCs with IC261 and gauged drug responses *in vitro* and *in vivo*. IC261 exhibited a strong cytotoxicity to LN229/GSCs, with an IC50 of 0.5 μM (Fig. 7A). This drug also robustly decreased the viability of GS9-6/NOTCH1 GSCs (Fig. 7B). Moreover, the growth of LN229/GSC xenograft tumors in immune-deficient mice was substantially inhibited by IC261 (Fig. 7C). The IC261-treated tumors were much smaller than those receiving vehicle DMSO at the end point (Fig. 7D). Further histological analysis confirmed the presence of malignant tumor cells in DMSO treatment group (Fig. 7E, top panel). Intriguingly, a large number of immune cells, but not tumor cells, were found in a tumor-like tissue in a mouse treated with IC261 (Fig. 7E, bottom panel). Hence, inhibiting CK1ε blocks GSCs’ growth *in vitro* and *in vivo*.

**Figure 8:**
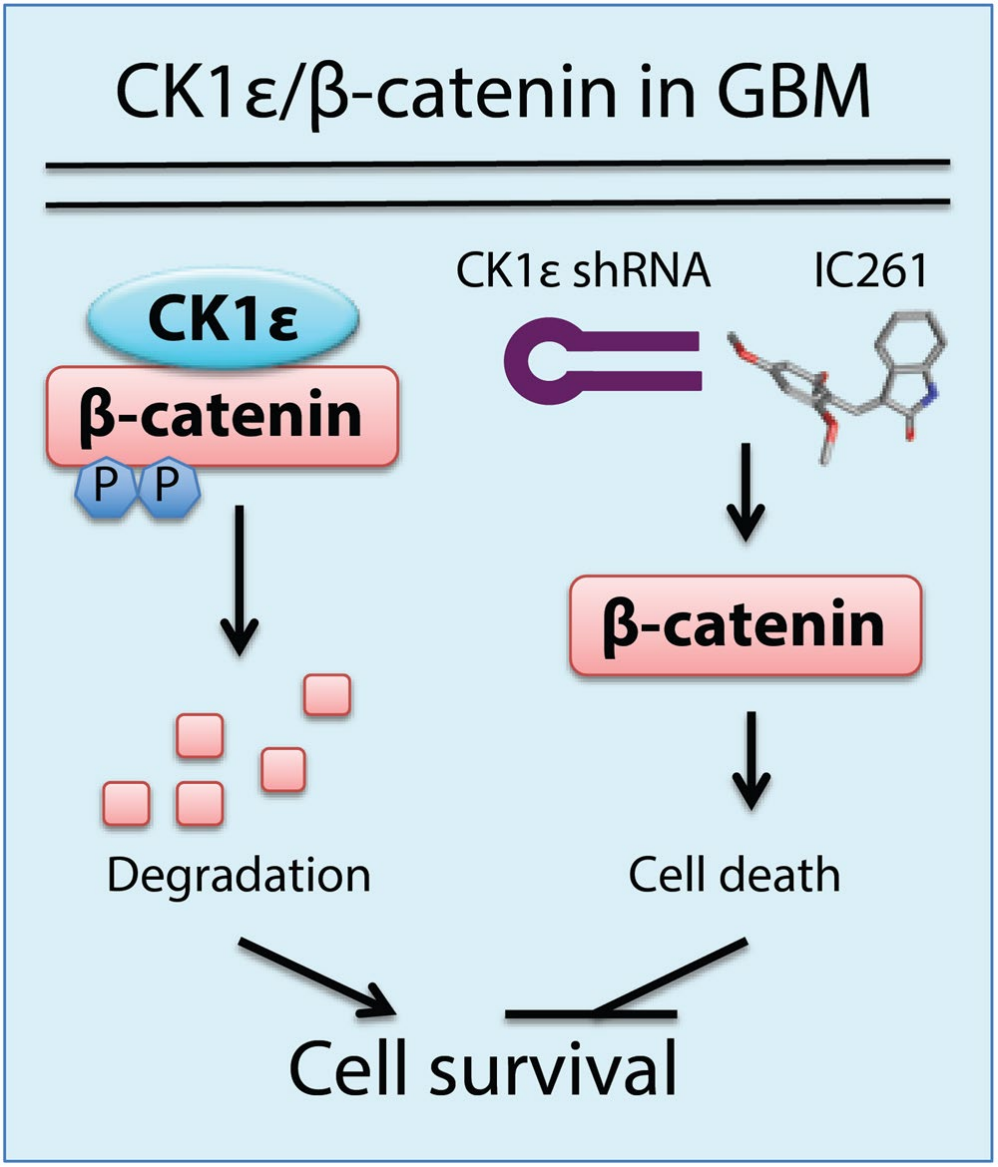
A noncanonical CK1ε/β-catenin signaling in GBM cell survival.

Taken together, results presented above demonstrate that a non-canonical CK1ε/β-catenin signaling pathway regulates GBM cell survival and targeting this pathway by shRNAs or chemical inhibitors represents an effective GBM treatment (Fig. 8).

## Discussion

The difficulty in treating GBM has motivated brain cancer researchers to search for novel and effective therapeutic options^38,39^. One current effort for this purpose involves genomic analyses e.g. DNA sequencing, gene expression profiling, as well as bisulfite sequencing of GBM. These large-scale analyses reveal potential genetic alterations that may be important for cancer cell survival, which still requires further investigation in cells and animal models^40,41^. Targeting known signaling molecules has also been interrogated in the clinic; however, the outcome of these treatments in GBM is still poor^42,43^. Therefore, new targets are needed. In this report, we demonstrate that a non-canonical CK1ε/β-catenin signaling pathway is essential for the survival of GBM cells as well as the self-renewal of GSCs (Fig. 8). Furthermore, inhibition of CK1ε blocks tumor growth *in vitro* and *in vivo*. Our results have important implications in the development of novel and effective therapies for GBM.

In addition to the identification of a novel therapeutic target for GBM, we also find that depletion of CK1ε activates β-catenin and subsequently induces growth inhibition and apoptosis in GBM and GSCs (Figs 4 and 5). While this finding is contradictory to the oncogenic role of canonical WNT/β-catenin in glioma^20,21^, induction of apoptosis by activated β-catenin has been well documented. For instance, over-expression, activation, or stabilization of β-catenin results in either cell death^44–47^ or delayed cell cycle^48^ in a variety of cells including stem cells. The oncogenic role of β-catenin in tumorigenesis including gliomagenesis may solely rely on the activity of canonical WNT/β-catenin signaling^21^. In our experimental settings, β-catenin is activated by the loss of CK1ε, a negative regulator of β-catenin signaling in the absence of WNT ligands. While no direct evidence was presented in this study, our results suggest a non-canonical CK1ε/β-catenin signaling, in which β-catenin is activated by CK1ε deficiency in the absence of WNT ligands and then transcriptionally activates a different set of genes to induce apoptosis and repress cell growth. Further investigation will elucidate the detailed mechanisms underlying β-catenin-activated apoptosis in GBM.

In the present study, we tested two commercially available CK1ε inhibitors IC261 and PF-4800567^35,36^. However, GBM cells displayed significant difference in responding to these two drugs, with PF-4800567 much less effective than IC261 (Fig. 6). These results are inconsistent with the fact that PF-4800567 is more selective in preventing CK1ε from activating circadian rhythm genes^35,36^. Nevertheless, our results shown in Fig. 6 suggest that PF-4800567 increases protein levels of CK1ε and concomitantly inactivates β-catenin. Hence, PF-4800567 fails to mimic the effect of CK1ε shRNA on β-catenin activation and the survival of GBM cells. It is therefore possible that CK1ε mediates activities of circadian rhythm genes and β-catenin through independent mechanisms. Because IC261 is an inhibitor of both CK1ε and CK1δ (36), the lack of specificity in selectively targeting CK1ε may limit its potential application in the clinic to treat GBM even though IC261 effectively blocks tumor growth *in vitro* and *in vivo* (Figs 6 and 7). Future studies will test the possibility of using IC261 as a GBM treatment and will also identify more CK1ε-selective inhibitors that are capable of activating β-catenin and inducing cell death in GBM cells.

## Methods

### Reagents

Chemical inhibitors of CK1ε include IC261 (Cayman Chemical) and PF-4800567 (Toris Bioscience). All chemicals were dissolved in dimethyl sulfoxide (DMSO) at stock concentrations ranging from 10 to 50 mM. The stock solutions were stored at −80 °C. When applied to cells, chemical inhibitors were directly diluted into the culture media at the desired final concentrations. Cells were typically incubated with inhibitors for four to seven days before further analyses.

### Cell lines

Nine human GBM cell lines (SF-295, SNB-75, LN-18, LN229, U87MG, SF-268, T98G, A172, and U251) were maintained in Dulbecco’s Modified Eagle Medium (DMEM, Life Technologies Corporation) supplemented with 10% fetal bovine serum (FBS; Atlas Biologicals, Inc.), 100 μg/ml of streptomycin, and 100 IU/ml of penicillin (Gibco). Two primary GBM cell lines (VTC-001 and VTC-002) were maintained in DMEM supplemented with 15% FBS (Peak Serum, Inc.) 100 μg/ml of streptomycin, and 100 IU/ml of penicillin (Gibco). Human astrocytes were cultured in AGM^TM^ astrocytes growth medium (Lonza). Two primary GBM cell lines were isolated as described previously^49^. Five human GSC lines (VTC-001/GSC, GS9-6/NOTCH1, LN-18/GSC, LN229/GSC, and U251/GSC) were prepared as described previously^18,31^ and were maintained as spheres in stem cell media, which includes DMEM, B-27® supplements (Life Technologies Corporation), 20 ng/ml FGF-2 (GenScript), and 20 ng/ml EGF (GenScript).

### Analysis of CK1 gene expression

Gene expression analysis was described previously with modifications^5,50^. The expression data of CK1 genes were retrieved from CellMiner, Oncomine, and The Human Protein Atlas. For the data from CellMiner, the arbitrary copy numbers of each individual CK1 mRNAs in four GBM cell lines were averaged. Error bars represent standard deviation of four sets of data. Fold changes of CK1 mRNAs in GBM over normal brain tissues and *P* values determining the significance of difference between two groups were retrieved from the Oncomine database. Immunofluorescence images of CK1ε in U251 cells and images of immunohistochemical analyses of CK1ε in cerebral cortex and high-grade glioma were obtained from the Human Protein Atlas.

### shRNA-mediated gene knockdown

shRNA-mediated knockdown was performed as previously described^50,51^. shRNAs of CK1ε and MELK (maternal embryonic leucine zipper kinase) were purchased from Thermo Fisher Scientific Inc. The vendor IDs of CK1ε or MELK shRNA were TRCN0000001837 and TRCN0000001645 respectively. The shRNA of β-catenin was purchased from Addgene (cat# 42544). Lentiviruses of individual shRNAs were made according to the manufacturer’s instruction. 5 × 10^5^ cells were seeded and transduced with lentiviruses of non-silencing (NS) shRNA or shRNAs of CK1ε, MELK, or β-catenin. Cells were then selected with 0.5–1.0 μg/ml puromycin for 7 days. Knockdown efficiency was assessed using immunoblotting.

### Immunoblotting

Immunoblotting was performed as described in our previous reports^5,18,30,50,52^. In brief, cells were lysed and total protein was quantified using the Bradford assay (Bio-Rad Laboratories Inc.). An equal amount of total protein (~50 μg) in each sample was loaded onto an SDS-PAGE gel. After transferring to PVDF membrane, the blot was incubated with antibodies. Antibodies of CK1ε, MELK, β-catenin, phospho-β-cateninThreonine41Serine45 (pβ-cateninT41S45), cleaved caspase 3 (c-CASP3), or GFAP (glia fibrillary associated protein), were purchased from Cell Signaling Technology. Antibody of active β-catenin was purchased from EMD Millipore Corporation. Anti-NOTCH1 and anti-β-actin (ACTB) antibodies were purchased from Sigma-Aldrich Co. LLC. Antibodies were diluted as follows: anti-CK1ε (1:500), anti-MELK (1:1000), anti-β-catenin (1:1000), anti-pβ-cateninT41S45 (1:200), anti-active β-catenin (1:500), anti-GFAP (1:1000), anti-c-CASP3 (1:1000), anti-NOTCH1 (1:1000), and anti-β-actin (1:5000). Images were taken using a ChemiDoc MP System (Bio-Rad Laboratories Inc.). The intensities of protein bands were quantified using Image J. The relative intensities were obtained by dividing the intensities of each protein to those of ACTB (loading control). For some experiments, the fold changes of proteins were obtained by dividing the intensities of experiments to those of the control.

### MTS viability assay

The MTS viability assay was described previously^18,50,52^. In brief, 1,000 to 2,000 cells were plated in a 96-well plate. Cells were then treated with DMSO and chemical inhibitors at the indicated doses. After 7 days, cell viability was measured using MTS according to manufacturer’s instruction (Promega). The absorbance at 490 nm was measured using a FilterMax F3 microplate reader (Molecular Devices, LLC). Percent cell viability was obtained by dividing the absorbance of treatment groups with those of untreated groups. IC50s were calculated using GraphPad Prism software.

### Caspase 3/7 activity assay

Caspase 3/7 activity was monitored using Caspase-Glo® 3/7 assay kit (Promega) and modified as previously described^50^. Cells were transduced with viruses containing NS or CK1ε shRNA. After puromycin selection, apoptosis was assessed using the Caspase-Glo® 3/7 assay kit per manufacturer’s instruction and cell number was determined using the MTS viability assay described above. The luminescence signal and the MTS absorbance were measured using a FilterMax F3 microplate reader. The relative caspase 3/7 activities were obtained by dividing luminescence readings with corresponding MTS absorbance readings. The fold changes of caspase3/7 activity were obtained by dividing relative caspase3/7 activities in CK1ε shRNA-treated cells to those in NS shRNA-treated cells.

### Luciferase reporter assay

Luciferase reporter assay was described previously^53,54^. The β-catenin reporter plasmids M50 Super 8x TOPFlash (abbreviated as TOPFlash) and M51 Super 8x FOPFlash (abbreviated as FOPFlash) were purchased from Addgene. Cells were transduced with viruses containing NS or CK1ε shRNA followed by transient transfection of β-catenin reporter plasmids using Effectene (QIAGEN). Luciferase activity was determined using a luciferase assay kit (Promega) based on manufacturer’s instruction. Cell numbers were quantified using the MTS viability assay. The reporter activities were obtained by normalizing the luminescence measurements with corresponding MTS readings.

### Stem cell self-renewal assay

Stem cell self-renewal assay (sphere formation assay) was described previously^18,31^. GSCs were plated in a 96 well plate at cell densities of 50 cells per well. GSCs were transduced with viruses containing NS, CK1ε, or MELK shRNA followed by puromycin selection as described previously. After 2 weeks, wells with spheres were counted and sphere pictures were taken using an inverted microscope with a 10X or 20X lens. Percentages of wells with spheres were obtained by dividing numbers of wells with spheres with numbers of wells plated.

### Mouse experiments

Mouse experiments were performed based on methods described previously^18,50,52,55^. All animal studies were approved by the Institutional Animal Care and Use Committee (IACUC) of Virginia Tech. All animal experiments were performed in accordance with the guidelines and regulations of IACUC. 2 × 10^5^ LN229/GSCs were mixed with Matrigel (Corning) and subcutaneously injected into BALB/c nude mice (Charles River Laboratories). 8 days after cell injection, mice were randomly divided into two groups to receive the following treatments: (1) DMSO; (2) 30 mg/kg IC261. Drugs were administered daily through intraperitoneal injection. During the treatment, tumors were measured daily using a caliper. On day 20, mice were euthanized and tumors were harvested because some tumors in the control group reached 1 cm in diameter. Tumor volumes (mm^3^) were calculated using the formula: (length × width × width)/2.

### Statistical analyses

Student’s *t* test was used to determine the difference of means between the control and treatment groups in gene expression analysis and mouse experiments.

## Electronic supplementary material

Supplemental materials

## Data Availability

All data are available for sharing upon request.

## References

[1] Ostrom QT (2016). CBTRUS Statistical Report: Primary Brain and Other Central Nervous System Tumors Diagnosed in the United States in 2009–2013. Neuro-oncology.

[2] Stupp R (2005). Radiotherapy plus concomitant and adjuvant temozolomide for glioblastoma. N Engl J Med.

[3] Weller M, Cloughesy T, Perry JR, Wick W (2013). Standards of care for treatment of recurrent glioblastoma–are we there yet?. Neuro-oncology.

[4] Omuro A, DeAngelis LM (2013). Glioblastoma and other malignant gliomas: a clinical review. JAMA: the journal of the American Medical Association.

[5] Varghese RT (2016). Survival kinase genes present prognostic significance in glioblastoma. Oncotarget.

[6] Zhang J, Yang PL, Gray NS (2009). Targeting cancer with small molecule kinase inhibitors. Nat Rev Cancer.

[7] Schittek B, Sinnberg T (2014). Biological functions of casein kinase 1 isoforms and putative roles in tumorigenesis. Mol Cancer.

[8] Yang Y, Xu T, Zhang Y, Qin X (2017). Molecular basis for the regulation of the circadian clock kinases CK1delta and CK1epsilon. Cell Signal.

[9] Brockschmidt C (2008). Anti-apoptotic and growth-stimulatory functions of CK1 delta and epsilon in ductal adenocarcinoma of the pancreas are inhibited by IC261 *in vitro* and *in vivo*. Gut.

[10] Yang WS, Stockwell BR (2008). Inhibition of casein kinase 1-epsilon induces cancer-cell-selective, PERIOD2-dependent growth arrest. Genome Biol.

[11] Kim SY (2010). CK1epsilon is required for breast cancers dependent on beta-catenin activity. Plos One.

[12] Ye LC (2015). Knockdown of Casein Kinase 1e Inhibits Cell Proliferation and Invasion of Colorectal Cancer Cells via Inhibition of the Wnt/beta-Catenin Signaling. J Biol Regul Homeost Agents.

[13] Rodriguez N (2012). Casein kinase I epsilon interacts with mitochondrial proteins for the growth and survival of human ovarian cancer cells. EMBO molecular medicine.

[14] Deng C (2017). Silencing c-Myc translation as a therapeutic strategy through targeting PI3Kdelta and CK1epsilon in hematological malignancies. Blood.

[15] Knippschild U (2014). The CK1 Family: Contribution to Cellular Stress Response and Its Role in Carcinogenesis. Front Oncol.

[16] Zhang S (2012). ROR1 is expressed in human breast cancer and associated with enhanced tumor-cell growth. Plos One.

[17] Nakano I (2008). Maternal embryonic leucine zipper kinase is a key regulator of the proliferation of malignant brain tumors, including brain tumor stem cells. J Neurosci Res.

[18] Murphy SF (2016). Connexin 43 Inhibition Sensitizes Chemoresistant Glioblastoma Cells to Temozolomide. Cancer Res.

[19] Cruciat CM (2014). Casein kinase 1 and Wnt/beta-catenin signaling. Current opinion in cell biology.

[20] Anastas JN, Moon RT (2013). WNT signalling pathways as therapeutic targets in cancer. Nature reviews. Cancer.

[21] Kaur N (2013). Wnt3a mediated activation of Wnt/beta-catenin signaling promotes tumor progression in glioblastoma. Molecular and cellular neurosciences.

[22] Amit S (2002). Axin-mediated CKI phosphorylation of beta-catenin at Ser 45: a molecular switch for the Wnt pathway. Genes Dev.

[23] Yost C (1996). The axis-inducing activity, stability, and subcellular distribution of beta-catenin is regulated in Xenopus embryos by glycogen synthase kinase 3. Genes Dev.

[24] Knippschild U (2005). The role of the casein kinase 1 (CK1) family in different signaling pathways linked to cancer development. Onkologie.

[25] Price MACKI (2006). there’s more than one: casein kinase I family members in Wnt and Hedgehog signaling. Genes Dev.

[26] Cheong JK, Virshup DM (2011). Casein kinase 1: Complexity in the family. Int J Biochem Cell Biol.

[27] Veeman MT, Slusarski DC, Kaykas A, Louie SH, Moon RT (2003). Zebrafish prickle, a modulator of noncanonical Wnt/Fz signaling, regulates gastrulation movements. Current biology: CB.

[28] Osuka S, Van Meir EG (2017). Overcoming therapeutic resistance in glioblastoma: the way forward. J Clin Invest.

[29] Parker NR, Khong P, Parkinson JF, Howell VM, Wheeler HR (2015). Molecular heterogeneity in glioblastoma: potential clinical implications. Frontiers in oncology.

[30] Pohlmann ES (2015). Real-time visualization of nanoparticles interacting with glioblastoma stem cells. Nano letters.

[31] Kanabur P (2016). Patient-derived glioblastoma stem cells respond differentially to targeted therapies. Oncotarget.

[32] Dirks PB (2008). Brain tumour stem cells: the undercurrents of human brain cancer and their relationship to neural stem cells. Philosophical transactions of the Royal Society of London. Series B, Biological sciences.

[33] Sheng Z, Chang SB, Chirico WJ (2003). Expression and purification of a biologically active basic fibroblast growth factor fusion protein. Protein Expr Purif.

[34] Ring A, Kim YM, Kahn M (2014). Wnt/catenin signaling in adult stem cell physiology and disease. Stem Cell Rev.

[35] Behrend L (2000). IC261, a specific inhibitor of the protein kinases casein kinase 1-delta and -epsilon, triggers the mitotic checkpoint and induces p53-dependent postmitotic effects. Oncogene.

[36] Walton KM (2009). Selective inhibition of casein kinase 1 epsilon minimally alters circadian clock period. The Journal of pharmacology and experimental therapeutics.

[37] Cheong JK (2011). IC261 induces cell cycle arrest and apoptosis of human cancer cells via CK1delta/varepsilon and Wnt/beta-catenin independent inhibition of mitotic spindle formation. Oncogene.

[38] Pridham KJ, Varghese RT, Sheng Z (2017). The Role of Class IA Phosphatidylinositol-4,5-Bisphosphate 3-Kinase Catalytic Subunits in Glioblastoma. Front Oncol.

[39] Grek CL (2018). Novel approach to temozolomide resistance in malignant glioma: connexin43-directed therapeutics. Curr Opin Pharmacol.

[40] Chen J, McKay RM, Parada LF (2012). Malignant glioma: lessons from genomics, mouse models, and stem cells. Cell.

[41] Riddick G, Fine HA (2011). Integration and analysis of genome-scale data from gliomas. Nature reviews. Neurology.

[42] Wen PY (2014). Phase I/II study of erlotinib and temsirolimus for patients with recurrent malignant gliomas: North American Brain Tumor Consortium trial 04-02. Neuro-oncology.

[43] Den RB (2013). A phase I study of the combination of sorafenib with temozolomide and radiation therapy for the treatment of primary and recurrent high-grade gliomas. Int J Radiat Oncol Biol Phys.

[44] Kim K, Pang KM, Evans M, Hay ED (2000). Overexpression of beta-catenin induces apoptosis independent of its transactivation function with LEF-1 or the involvement of major G1 cell cycle regulators. Mol Biol Cell.

[45] Ming M (2012). Activation of Wnt/beta-catenin protein signaling induces mitochondria-mediated apoptosis in hematopoietic progenitor cells. J Biol Chem.

[46] Edlund S (2005). Interaction between Smad7 and beta-catenin: importance for transforming growth factor beta-induced apoptosis. Mol Cell Biol.

[47] Zimmerman ZF, Kulikauskas RM, Bomsztyk K, Moon RT, Chien AJ (2013). Activation of Wnt/beta-catenin signaling increases apoptosis in melanoma cells treated with trail. Plos One.

[48] Kimura T (2006). The stabilization of beta-catenin leads to impaired primordial germ cell development via aberrant cell cycle progression. Dev Biol.

[49] Adams MN (2015). EGF inhibits constitutive internalization and palmitoylation-dependent degradation of membrane-spanning procancer CDCP1 promoting its availability on the cell surface. Oncogene.

[50] Pridham KJ (2018). PIK3CB/p110beta is a selective survival factor for glioblastoma. Neuro Oncol.

[51] Guo S (2018). A large-scale RNA interference screen identifies genes that regulate autophagy at different stages. Sci Rep.

[52] Guo S (2015). A rapid and high content assay that measures cyto-ID-stained autophagic compartments and estimates autophagy flux with potential clinical applications. Autophagy.

[53] Sheng Z, Wang SZ, Green MR (2009). Transcription and signalling pathways involved in BCR-ABL-mediated misregulation of 24p3 and 24p3R. Embo J.

[54] Sheng Z (2010). A genome-wide RNA interference screen reveals an essential CREB3L2-ATF5-MCL1 survival pathway in malignant glioma with therapeutic implications. Nature medicine.

[55] Li T (2015). A New Interleukin-13 Amino-Coated Gadolinium Metallofullerene Nanoparticle for Targeted MRI Detection of Glioblastoma Tumor Cells. Journal of the American Chemical Society.

