# Supplementary material for: Casein Kinase 1 Epsilon Regulates Glioblastoma Cell Survival
Source: Sci Rep. 2018 Sep 11;8:13621. doi: 10.1038/s41598-018-31864-x (PMC6134061; doi:10.1038/s41598-018-31864-x)

Casein Kinase 1 Epsilon Regulates Glioblastoma Cell Survival

Robin T Varghese, Sarah Young, Lily Pham, Yanping Liang, Kevin J Pridham, Sujuan Guo, Susan Murphy, Deborah F Kelly, and Zhi Sheng

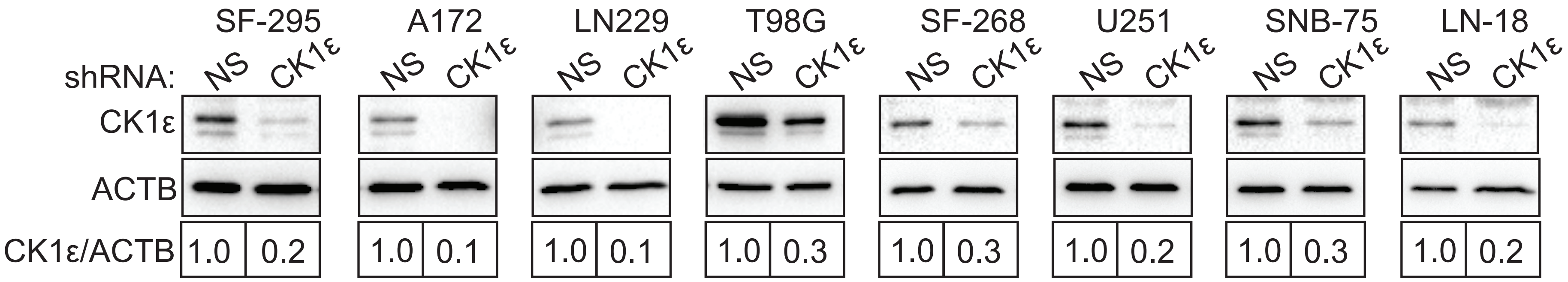

Figure S1. Knockdown of CK1ε in GBM cell lines. GBM cells were treated with non-silencing (NS) or CK1ε shRNA. Protein levels of CK1ε were analyzed using immunoblotting. ACTB (β-actin) is the loading control. Band intensities were quantified using Image J.

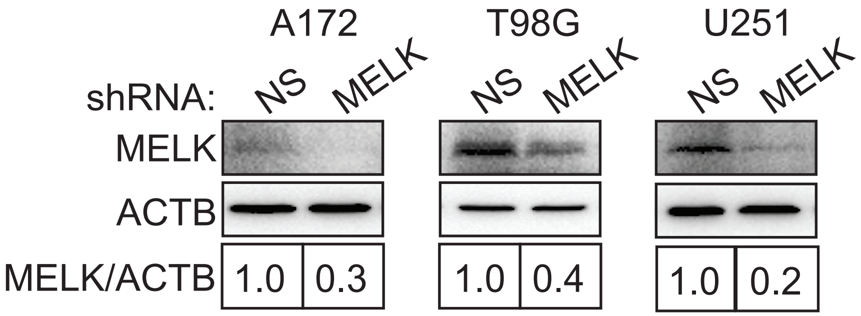

Figure S2. Knockdown of MELK in GBM cell lines. GBM cells were treated with non-silencing (NS) or MELK shRNA. Protein levels of MELK were analyzed using immunoblotting. ACTB (β-actin) is the loading control. Band intensities were quantified using Image J.

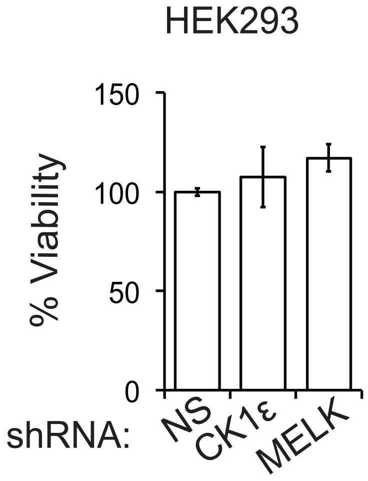

Figure S3. Effect of CK1ε and MELK Knockdown on the viability of HEK293. HEK293 cells were treated with non-silencing (NS) or shRNA of CK1ε or MELK. Cell viability was monitored the MTS assay.

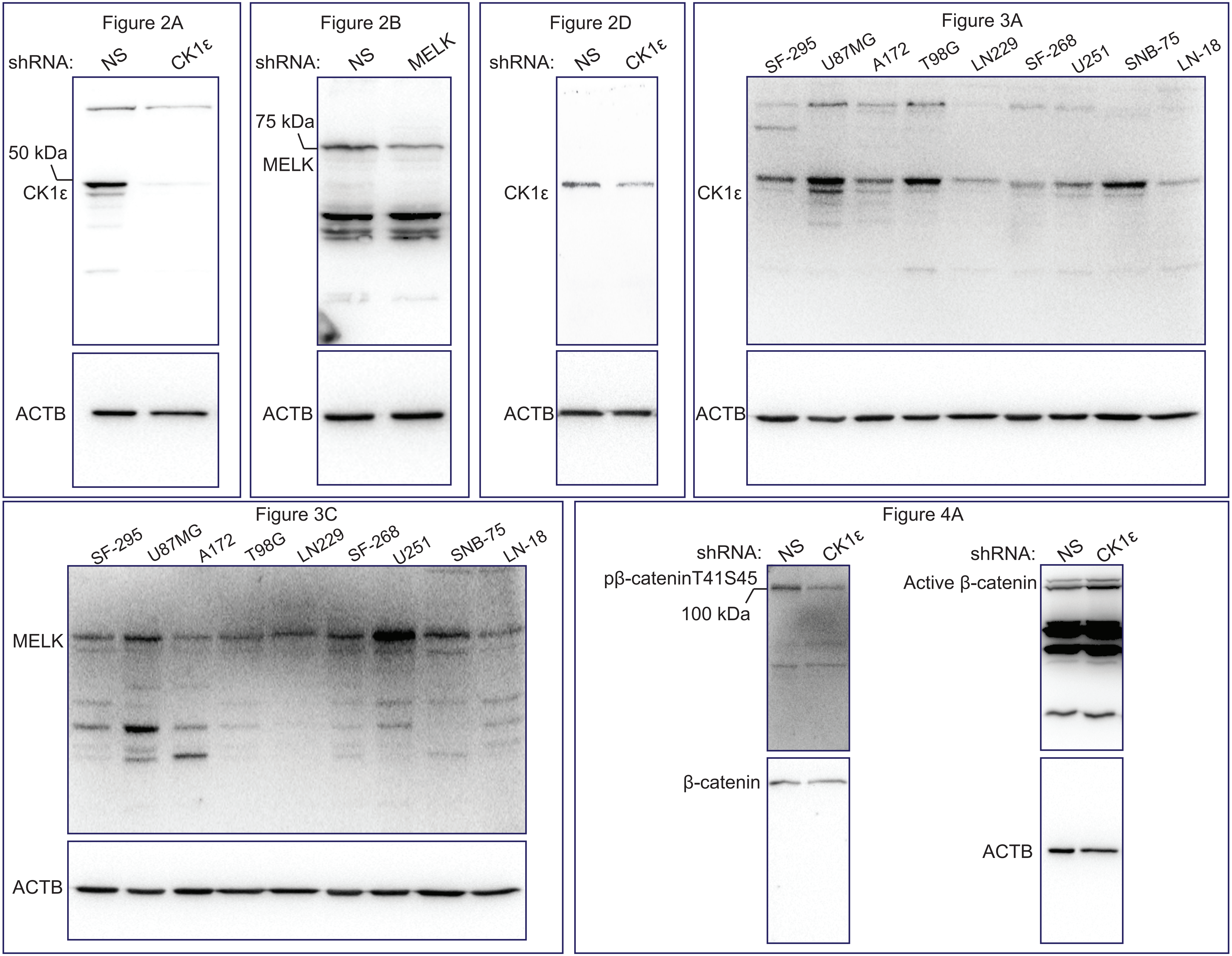
Figure 2A
shRNA: NS CK1ε
50 kDa
CK1ε
ACTB
Figure 2B
shRNA: NS MELK
75 kDa
MELK
ACTB
Figure 2D
shRNA: NS CK1ε
CK1ε
ACTB
Figure 3A
SF-295 U87MG A172 T98G LN229 SF-268 U251 SNB-75 LN-18
CK1ε
ACTB
Figure 3C
SF-295 U87MG A172 T98G LN229 SF-268 U251 SNB-75 LN-18
MELK
ACTB
Figure 4A
shRNA: NS CK1ε
pβ-cateninT41S45
100 kDa
β-catenin
shRNA: NS CK1ε
Active β-catenin
ACTB

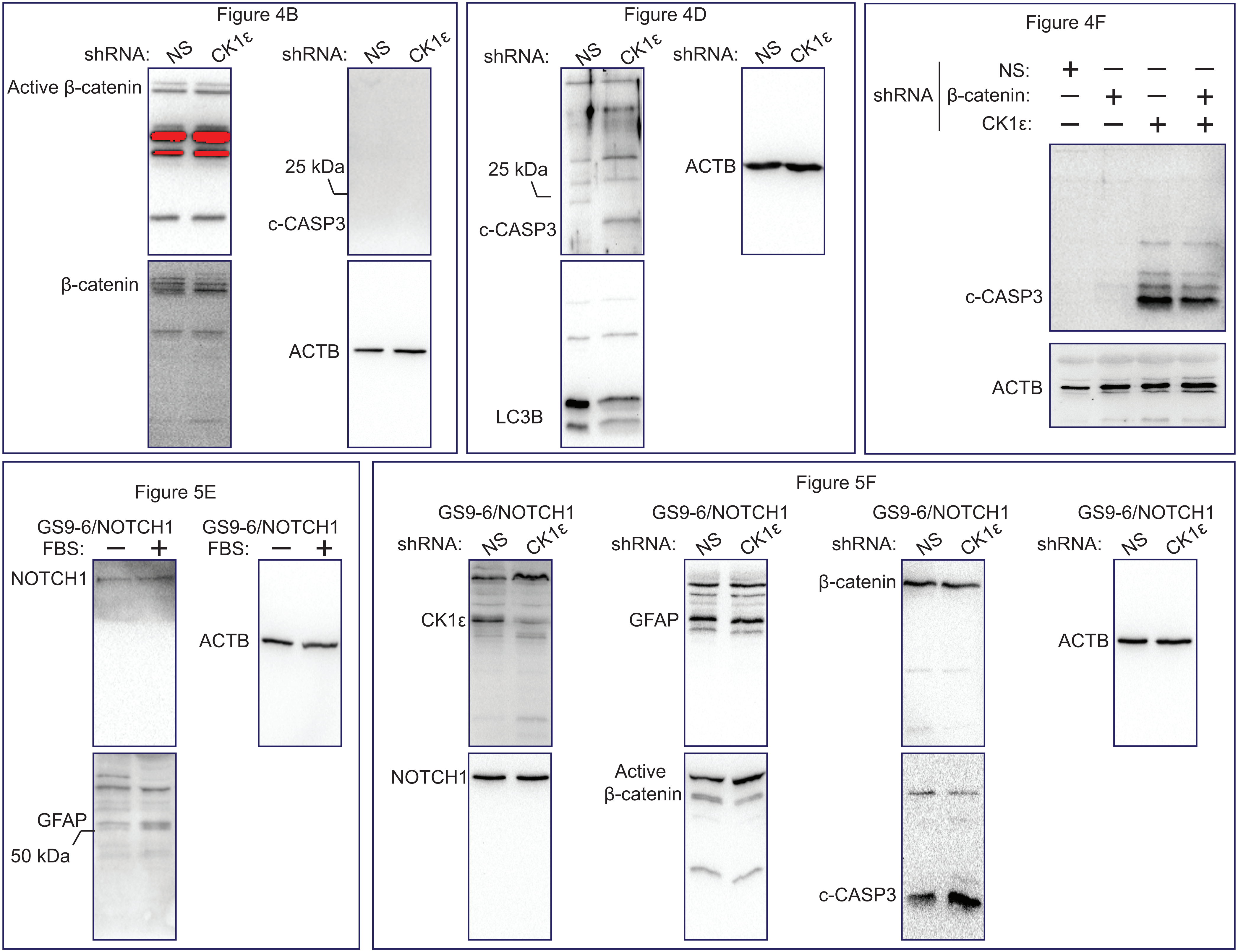

Figure 4B
shRNA: NS CK1ε
Active β-catenin
β-catenin
shRNA: NS CK1ε
25 kDa
c-CASP3
ACTB
Figure 4D
shRNA: NS CK1ε
25 kDa
c-CASP3
LC3B
shRNA: NS CK1ε
ACTB
Figure 4F
shRNA
NS: + − − −
β-catenin: − + − +
CK1ε: − − + +
c-CASP3
ACTB
Figure 5E
GS9-6/NOTCH1
FBS: − +
NOTCH1
GFAP
50 kDa
GS9-6/NOTCH1
FBS: − +
ACTB
Figure 5F
GS9-6/NOTCH1
shRNA: NS CK1ε
CK1ε
NOTCH1
GS9-6/NOTCH1
shRNA: NS CK1ε
GFAP
Active β-catenin
GS9-6/NOTCH1
shRNA: NS CK1ε
β-catenin
c-CASP3
GS9-6/NOTCH1
shRNA: NS CK1ε
ACTB

Figure 4F

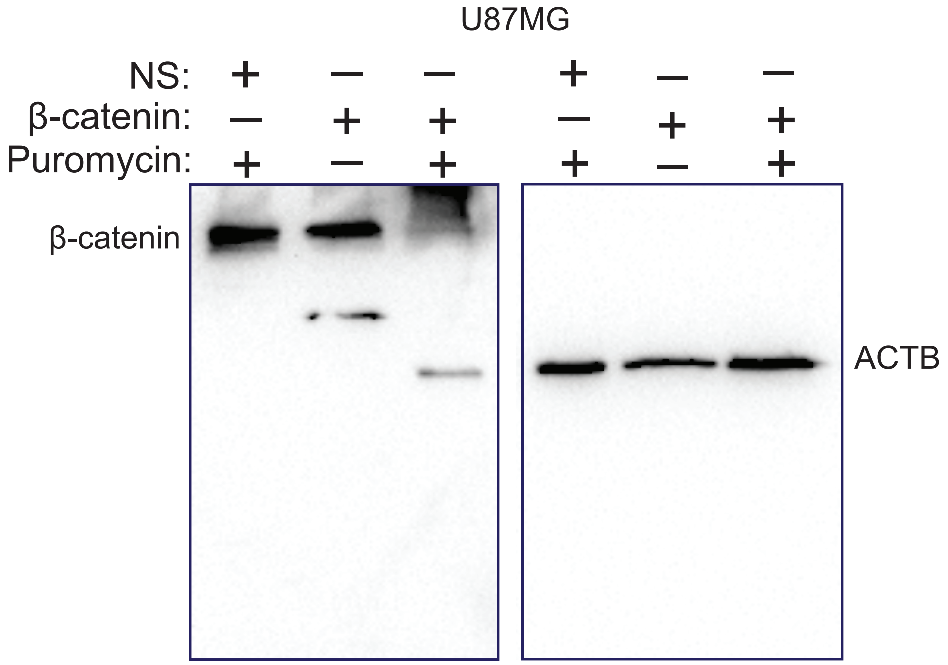

Figure 6D

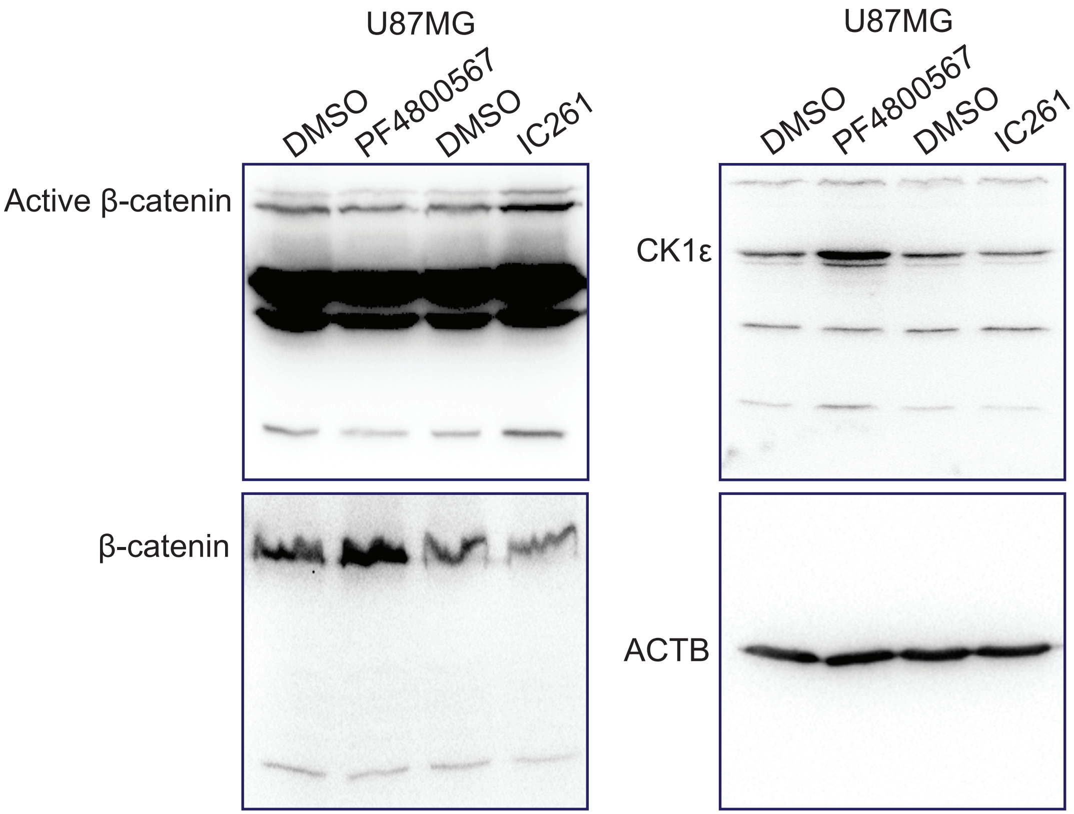

Supplement: Supplementary file 1 — Supplemental materials [file 41598_2018_31864_MOESM1_ESM.pdf]
